# Association between plural legal systems and sexual and reproductive health outcomes for women and girls in Nigeria: A state-level ecological study

**DOI:** 10.1371/journal.pone.0223455

**Published:** 2019-10-09

**Authors:** Terry McGovern, Monique Baumont, Rachel Fowler, Valentina Parisi, Sonia Haerizadeh, Eka Williams, Samantha Garbers

**Affiliations:** 1 Heilbrunn Department of Population and Family Health, Columbia University Mailman School of Public Health, New York, New York, United States of America; 2 Department of Sociomedical Sciences, Columbia University Mailman School of Public Health, New York, New York, United States of America; 3 Independent consultant, São Paulo, Brazil; 4 Independent consultant, Johannesburg, South Africa; University of Cape Coast, GHANA

## Abstract

Nigeria has a plural legal system in which various sources of law govern simultaneously. Inconsistent and conflicting legal frameworks can reinforce pre–existing health disparities in sexual and reproductive health (SRH). While previous studies indicate poor SRH outcomes for Nigerian women and girls, particularly in Northern states, the relationship between customary and religious law (CRL) and SRH has not been explored. We conducted a state-level ecological study to examine the relationship between CRL and SRH outcomes among women in 36 Nigerian states and the Federal Capital Territory of Abuja (n = 37), using publicly available Demographic and Health Survey data from 2013. Indicators were guided by published research and included contraception use among married women, total fertility rate, median age at first birth, receipt of antenatal care, delivery location, and comprehensive knowledge of HIV. To account for economic differences between states, crude linear regression models were compared to a multivariable model, adjusting for per capita GDP. All SRH outcomes, except comprehensive knowledge of HIV, were statistically significantly more negative in CRL states compared to non–CRL states, even after accounting for state–level GDP. In CRL states in 2013, compared to non–CRL states, the proportion of married women who used any method of contraception was 22.7 percentage points lower ([95% CI: −15.78 –−29.64], p<0.001), a difference that persisted in a model adjusting for per capita GDP (b[adj] = −16.15, 95% CI: [−8.64 –−23.66], p<0.001.). While this analysis of retrospective state-level data found robust associations between CRL and poor SRH outcomes, future research should incorporate prospective individual-level data to further elucidate these findings.

## Introduction

Many countries implement civil laws that reflect or adhere to international human rights standards. However, there are many oft-overlooked complications to the domestication of human rights instruments. More than half of the countries in Africa have plural legal systems, in which multiple sources of law govern simultaneously, fueling contradictions and inconsistencies in both the provision and interpretation of the law [1, 2]. While in some areas customary and religious law (CRL) may be more creative and solution-oriented than civil or international law remedies, CRL can also undermine constitutional and statutory provisions and permit discriminatory cultural and religious practices to persist [3]. This is particularly true in the realm of gender justice and sexual and reproductive health and rights [2, 3]. National governments have made exceptions to international covenants, such as the Convention on the Rights of the Child (CRC) and Convention on the Elimination of Discrimination against Women (CEDAW), on the grounds that they violate religious edicts or customary practices, limiting the impact of these international standards [4]. Many countries also make constitutional exceptions for communities in which CRL apply or they exempt matters pertaining to family or privacy from civil or international law. Treaty-monitoring bodies have consistently raised concern about the co-existence and use of discriminatory CRL alongside codified law [5–7].

CRL has been found to uphold practices that discriminate against women and undermine gender equality [1, 8–9]. Adherence to CRL has been linked to high rates of child marriage, decreased female autonomy, and limited access to justice for women and girls [10]. A multi–country study mapping the presence of plural legal systems with the percentage of women aged 20–24 who were married or in a union before the age of 18 identified a clear pattern between the presence of plural legal systems and high levels of child marriage [11]. An analysis of 162 countries found that the odds of child marriage are approximately four times greater in the presence of a plural legal system than without [11].

Existing research on how CRL affects the sexual and reproductive health (SRH) of women and girls has primarily focused on child marriage. An analysis of 115 low- and middle-income countries found that countries with strict minimum age of marriage laws (18 years) experience the greatest declines in adolescent fertility rates compared to countries with legal exceptions or no minimum-age marriage laws [12]. A study of 12 countries in Sub-Saharan Africa similarly found that the prevalence of teenage childbearing in countries with consistent marriage laws—defined as countries with a minimum age of 18 or older without exceptions under CRL for age marriage, age of marriage with parental consent, and age of sexual consent—was 25% lower than in countries with inconsistent laws [13]. Child marriage may also impact SRH through increased school drop–out rates [14–16]. Lower education levels have been strongly associated with low contraceptive use among women and high fertility rates in multiple developing countries [17]. In addition to child marriage, lack of decision–making autonomy has been found to decrease utilization of antenatal care and health facility delivery among women and girls [18–21].

## CRL and SRH in Nigeria

Nigeria is characterized by religious, ethnic, and legal pluralism. Adherence to CRL in Northern regions of Nigeria is closely linked to the country’s history of regionalism. Upon gaining independence in 1960, Nigeria established a constitution in which three regions—including northern, eastern, and western regions—would operate with unique laws and governments distinct from a federal system [22]. This configuration facilitated religious and ethnic diversity, reinforced regional differences, and dampened national identity. Regional frictions over the control of state resources mounted in the 1960s, prompting several military coups and a civil war [23].

In response to increased centralization after the civil war, Northern states further embraced regional autonomy through adherence to Islamic or Sharia law in both civil and criminal matters. Between 1999 and 2001, 12 Northern states reinstated Islamic criminal law in their jurisdictions [24]. In present-day Nigeria, the primary sources of law include: (1) the Constitution, which has binding force on all authorities and persons throughout the country; (2) legislation, of which each of the 36 states and the Federal Capital Territory, Abuja, have its own laws; (3) English law; (4) Customary law (ethnic, non–Islamic) that applies to members of different ethnic groups and is particularly dominant in the area of personal and family relations; and (5) Islamic law, often codified in Northern states [25]. Of Nigeria’s three Northern regions (North Central, North East, Northwest) and three Southern regions (South East, South South, South West), all states with codified CRL are located in Northern regions. As of 2013, 12 Northern states followed the Sharia Penal Code, four of which (Niger, Gombe, Kaduna, and Katsina) also amended the existing 1960 Nigerian Penal Code with provisions of Sharia criminal law [26].

Aspects of CRL have undermined efforts to improve SRH and gender equality through law and/or policy on the age of marriage and age of sexual consent, and the promotion of harmful practices. Under civil law, the 2003 Child Rights Act (CRA) establishes the age of marriage as 18 for both sexes. However, individual states must incorporate the act into their legislation in order to give it force [27]. As of 2016, only 26 of Nigeria’s 36 states had adopted the CRA and many made exceptions that maintain the age of marriage at 16 or at the age of “puberty” [28]. In states that have not adopted the CRA, the age of marriage is outlined by state level legislation, customary law, or Islamic law, which can set the age as young as nine years old or by the “age of puberty” [28].

In Nigerian states that have adopted the CRA without exception or the 2013 Sexual Offenses Act, the age of sexual consent for males and females is 18. However, due in part to opposition by the Supreme Council for Sharia, many Northern states have not adopted either of these acts, resulting in variation in the age of sexual consent by state and by the parties’ religion and sex [28]. In some states, for females, the age of sexual consent can be defined as “puberty” and/or age 15, 18, or age of marriage in other states [29, 30].

Polygamy is authorized and widely practiced under both customary and Islamic law. Nearly one-third of Nigerian women are in polygamous unions [31]. The fourth country report on the implementation of the African Charter on Human and People’s Rights in Nigeria noted that many CRLs in the country continue to support additional practices that have been shown to undermine gender equality including early marriage, early and unspaced child bearing, female genital mutilation, widowhood rites, and dis-inheritance [32].

Despite legal commitments to improving SRH, the status of SRH among women and girls in Nigeria remains poor. Nigeria has the second largest HIV epidemic in the world, with women making up more than half of people living with HIV [33]. Nigeria has persistently high maternal and perinatal mortality rates, low contraceptive prevalence, high incidence of unsafe abortions, and high levels of female genital cutting [34, 35]. Approximately 24% of women in Nigeria have reported ever experiencing intimate partner violence [36]. In 2013, Nigeria accounted for approximately 14% of the global burden of maternal mortality [37].

Prior research has shown that Northern regions of Nigeria have consistently had higher rates of child marriage, lower contraceptive use, lower receipt of antenatal care, fewer births delivered in a health facility, and higher total fertility rates and adolescent fertility rates than Southern regions [35, 37, 38]. In 2008, 48% of girls in the Northwest region were married by age 15 and 78% were married by age 18 [39]. On average, women in the Southeast region married more than seven years later than women in the Northwest region (22.8 years and 15.2 years, respectively) [39]. Levels of formal educational attainment are also lower in Northern Nigeria compared to Southern regions [38]. In many countries, women’s level of education has positively correlated with knowledge of HIV/AIDS [40].

Consistently poor SRH outcomes in Northern states compared to Southern states begets an interrogation of the relationship between CRL and SRH outcomes in Nigeria. Overall, the literature on the impacts of CRL on the health and well-being of women and girls remains largely theoretical [8, 9, 41]. While a few studies in other low- and middle-income countries have documented the impact of inconsistent marriage laws on SRH outcomes, these studies did not assess impacts on a broad range of SRH outcomes [12, 13]. Studies regarding determinants of SRH outcomes in Nigeria have primarily focused on the impact of individual and household factors on single SRH outcomes such as contraceptive use [42]. Researchers have noted that individual and household factors are insufficient to explain variations in contraceptive use and other SRH outcomes in Nigeria, and have identified a need for additional research on contextual factors [42].

To address the lack of statistical analysis on the relationship between CRL and SRH outcomes in the evidence base, we conducted a state-level ecological study utilizing publicly available data from the Demographic and Health Surveys (DHS) Program in Nigeria in 2013. We assessed the impact of CRL on a range of SRH outcomes that past research indicates have been impacted by CRL. We utilized six standardized and widely accepted indicators for these outcomes from the World Health Organization and MEASURE Evaluation [43, 44]. While we theorize that other areas of SRH beyond these six indicators are affected by CRL, further research is required to establish the link. We aim to examine the relationship between CRL and SRH outcomes in Nigeria and inform future research by suggesting potential pathways by which CRL negatively impacts SRH in this context.

## Materials and methods

### Data sources

These ecologic analyses make use of publicly available state-level data from the Demographic and Health Survey (DHS) program, which collects nationally representative health data on households, men, women, and children and generates reports with country–specific and comparative summary data. Publicly available aggregated DHS data (https://dhsprogram.com/Data/) from 2013 were used to examine SRH indicators for all 36 states plus the Federal Capital Territory of Abuja (n = 37). The survey used a stratified three-stage cluster sampling design, utilizing enumeration areas from the 2006 census as the primary sampling unit. The sample was stratified to ensure sufficient urban and rural representation in each state. In the first stage, 893 localities were selected with probability proportional to size. One enumeration area (EA) was then randomly selected from most of the selected localities, with more than one EA selected in larger localities, for a total of 904 clusters. From a list of households, 45 were selected in each urban and rural cluster through equal probability systematic sampling, for a total of 40,680 households. All women aged 15–49 who were usual members of the selected households or who spent the night before the survey in the household were eligible for individual interviews. Data for this study was retrieved from DHS reports generated from the woman’s questionnaire, in which 38,948 women ages 15–49 were interviewed on background characteristics, reproductive history, childhood mortality, knowledge and use of family planning methods, fertility preferences, antenatal, delivery, and postnatal care, female empowerment, and other health issues. The underlying data retrieved from DHS reports were weighted to ensure representativeness of survey results.

### Exposure variable

**Fig 1:**
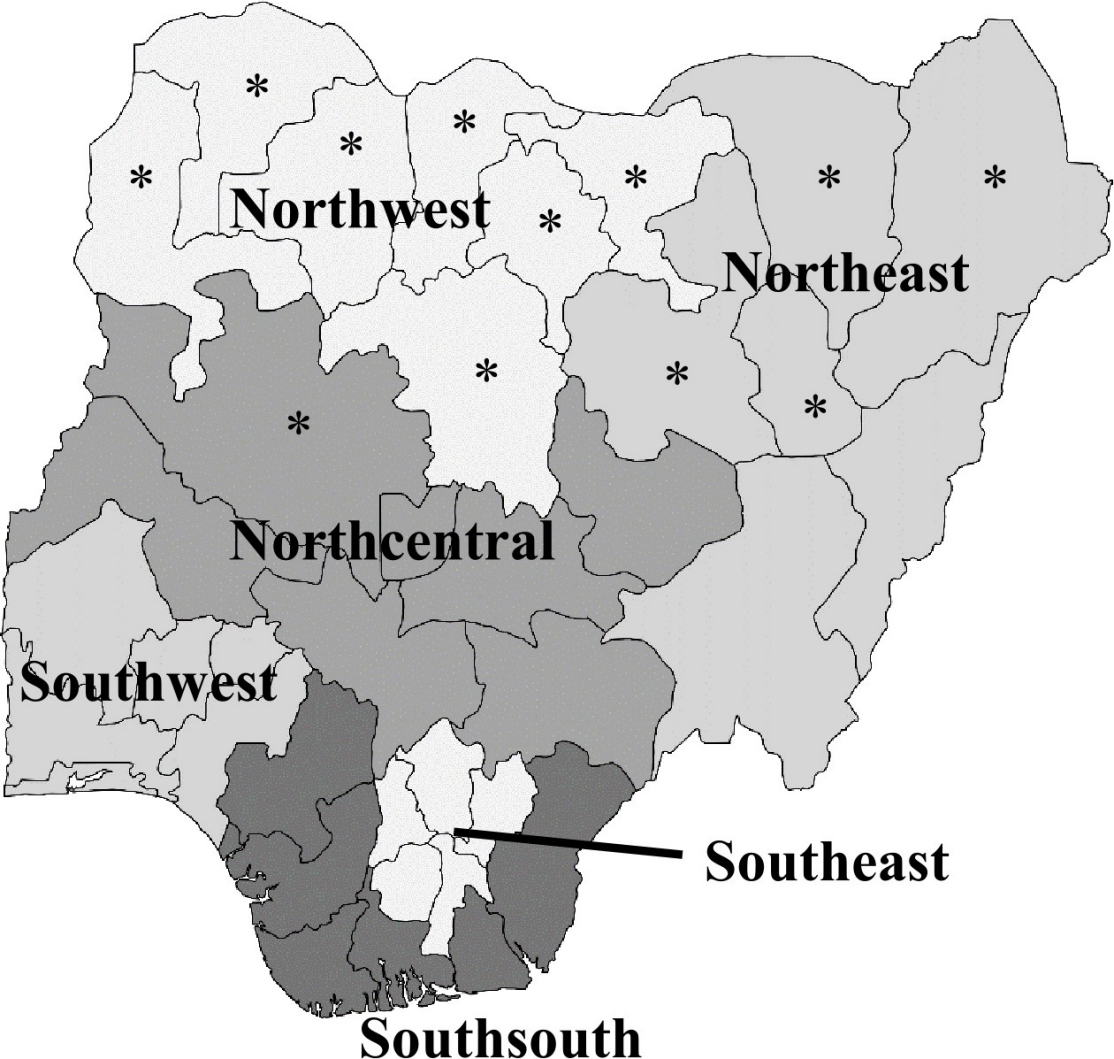
Map of Nigeria by region, 2013. Note: Classification of states by region. States with * were classified as having CRL in 2013. Northwest Region (7 of 7 states with CRL):*Jigawa, *Kaduna, *Kano, *Katsina, *Kebbi, *Sokoto, *Zamfara; Northeast Region (4 of 6 states with CRL): *Bauchi, *Borno, *Gombe, *Yobe, Adamawa, Taraba. Northcentral Region (1 of 7 states with CRL): *Niger, Kwara, Kogi, Federal Capital Territory, Nasarawa, Benue, Plateau; Southwest Region (0 of 6 states with CRL): Ekiti, Lagos, Ogun, Ondo, Osun, Oyo; Southeast Region (0 of 5 states with CRL): Abia, Anambra, Ebonyi, Enugu, Imo; Southsouth Region (0 of 6 states with CRL): Akwa Ibom, Bayelsa, Cross River, Delta, Edo, Rivers.

A dichotomized (yes/no) variable was created to designate whether each state had CRL, defined as codified customary and/or religious law. Out of 37 states, 12 states were identified as having CRL in 2013. Fig 1 presents the classification of states as CRL or non–CRL.

### Outcome variables

Six indicators were selected to assess SRH outcomes for women across the areas of family planning, maternal health, fertility, and HIV/AIDS. Selection of these indicators was guided by prior published work on the mechanisms through which CRL may adversely affect SRH in other countries: 1) early marriage and childbearing; 2) limited educational opportunities (which may affect knowledge of HIV); and 3) limited decision-making autonomy (which may affect care-seeking for contraception, antenatal care, and health facility deliveries) [10, 14–16, 20–21]. To operationalize these outcomes, we used standardized SRH indicators from the World Health Organization (contraceptive use, antenatal care, health facility delivery, and total fertility rate) and MEASURE Evaluation (median age at first birth), for which state-level DHS data was available in 2013 [43, 44]. While maternal mortality ratio (MMR) is considered a key SRH indicator, this indicator was excluded since state-level MMR was not available.

The indicator for family planning was the proportion of currently married women ages 15–49 who currently use any contraception method. We selected the indicator for married women to align with the MEASURE Evaluation conventions in reporting contraceptive prevalence. Maternal health indicators included: the proportion of births in the five years preceding the survey that were delivered in a health facility, and the proportion of women who received any antenatal care. Receipt of any antenatal care was operationalized as the number of women who reported at least one antenatal care visit for their most recent live birth, among women ages 15–49 who had a live birth in the five years preceding the survey. Fertility indicators included: total fertility rate, and the median age at first birth among women ages 25–49. The DHS report excludes women ages 15–24 from median age at first birth to ensure that half of the women have already had a birth when surveyed [35]. The indicator for HIV/AIDS was comprehensive knowledge of HIV among women ages 15–49, defined as the percentage of women 15–49 who correctly identified both major ways of preventing sexual transmission of HIV, said that a healthy-looking person can have the AIDS virus, and who rejected all three major misconceptions about HIV transmission or prevention.

### Data analysis

A crude linear regression model was run in IBM SPSS Statistics for Windows, Version 25.0 (IBM Corp., Armonk, NY, USA) to assess the linear association between each of the six SRH outcomes and status as a CRL or non–CRL state in 2013. For the SRH indicators examined, we used a linear categorization because it allows the analyses to pick up differences across the distribution of the indicators, not just above and below a dichotomous-level threshold. The Nigeria DHS does not include indicator objectives or benchmarks that would provide a rationale for dichotomizing the outcomes. For each association that was statistically significant in the crude model, we accounted for differences in baseline wealth levels between Northern and Southern states by re-running the regression models and including the per capita GDP in the model as a potential confounder. State–level GDP data from 2010 published by the National Bureau of Statistics was used to adjust for economic differences between states. The level of significance for all analyses was set a priori at 0.05.

## Results

In 2013, states with CRL had statistically significantly worse SRH outcomes compared to non–CRL states for five of the six outcomes examined (Table 1). In CRL states in 2013, 47.89% of women ages 15–49 who gave birth in the last five years received any antenatal care during their most recent birth, compared to 85.44% in non–CRL states, a 37.55 percentage point difference ([95% CI: −26.17 –−48.94], p<0.001). Similar statistically significant gaps by CRL status were observed for the proportion of married women who used any method of contraception (22.71 percentage points lower in CRL vs. non–CRL states, [95% CI: [−15.78 –−29.64], p<0.001), proportion of births delivered in a facility (47.66 percentage points lower [95% CI: 34.81–60.51], p<0.001), total fertility rate (1.96 higher, [95% CI: 1.34–2.59], p<0.001), and median age at first birth (3.37 years younger, [95% CI: - 2.29 –- 4.44], p<0.001). For comprehensive knowledge of HIV, approximately 24.78% of respondents demonstrated comprehensive knowledge in CRL states, compared to 27% in non-CRL states. This difference was not statistically significant (p = 0.632).

**Table 1:** SRH outcomes among CRL and non-CRL states (2013): Crude and adjusted linear regression analyses (n = 37).

	Non-CRL States (n = 25 states)	CRL States (n = 12 states)	Crude Model: CRL vs. not CRL Parameter estimate (95% CI)	Adjusted: CRL vs. not CRL + Econ^a^ Parameter estimate (95% CI)
Married women who use any method of contraception (%)	26.38	3.68	b = −22.71 (−15.78 –−29.64) p < .001	b = −16.15 (−8.64 –−23.66) p < .001
Women who received any antenatal care (%)	85.44	47.89	b = −37.55 (−26.17 –−48.94) p < .001	b = −35.79 (−21.29 –−50.30) p < .001
Births delivered in a health facility (% of all births)	62.13	14.47	b = −47.66 (−34.81 –−60.51) p < .001	b = −45.26 (−28.90 –−61.62) p < .001
Total fertility rate	4.74	6.71	b = 1.96 (1.34–2.59) p < .001	b = 1.48 (0.72–2.23) p < .001
Median age at first birth among women ages 25–49	21.74	18.37	b = −3.37 (−2.29 –−4.44) p < .001	b = −2.81 (−1.54 –−4.07) p < .001
Comprehensive knowledge of HIV (%)	27.00	24.78	b = −2.21 (−11.50–7.07) p = .632	Not run

^a^ Adjusted for 2010 state–level GDP per capita from National Bureau of Statistics

The Northern regions, where CRL states are located, have significantly higher poverty rates than Southern regions [45], a potential confounder. Adjusted models were run for all outcomes except for comprehensive knowledge of HIV, which was not statistically significantly associated with CRL status in the crude model. Even after taking into account each state’s GDP in 2010, CRL states had significantly worse SRH outcomes compared to non–CRL states. In all five adjusted models, parameter estimates decreased, yet the relationship between CRL and each SRH outcome remained statistically significant. Compared to non–CRL states, there were statistically significant differences, in the same direction, by CRL status observed for the proportion of married women who used any method of contraception (b[adj.] = −16.15 [95% CI: −8.64 –−23.66], p<0.001), the proportion of women receiving any antenatal care (b[adj.] = −35.79 [95% CI: −21.29 –−50.30], p<0.001), the proportion of births in a health facility (b[adj.] = −45.26 [95% CI: −28.90 –−61.62], p<0.001), total fertility rate (b[adj.] = 1.48 [95% CI: 0.72–2.23], p<0.001), and median age at first birth (b[adj.] = 2.81 [95% CI: 1.54–4.07], p<0.001), adjusting for per capita GDP.

## Discussion

Our analysis indicates persistent and significantly worse SRH outcomes in CRL states, compared to states without CRL. When differences between states with and without CRL were explored through linear regression models, CRL states had statistically significantly worse performance on all SRH indicators examined except for comprehensive knowledge of HIV. These findings support previous studies, which suggest that CRL reinforces practices and traditions that discriminate against women and negatively affect their health [8–9, 41].

The lack of statistically significant relationship between CRL and HIV knowledge in Nigeria is likely reflective of substantial state-level heterogeneity of HIV knowledge and prevalence, indicating that a complex combination of local factors influence HIV knowledge [46, 47]. For instance, Christian and Muslim practices and doctrines vary widely between and within states, and often have both positive and negative influences on HIV knowledge, risk factors, and practices [48]. Future studies should explore whether CRL impacts other factors related to HIV, such as AIDS-related stigma or access to treatment and preventive services.

Since rates of poverty are much higher in Northern states compared to Southern states, with more than 66% of impoverished Nigerians living in the North in 2014, economic differences between states was a potential confounder [45]. While there was some confounding by economic conditions of the states, economic differences were insufficient to explain statistically significant differences detected between CRL and non–CRL states. Even after taking this into account, significant differences between CRL and non–CRL states remained, suggesting that the relationship between CRL and adverse SRH in Nigeria is robust.

Our results corroborate multiple theoretical frameworks that suggest plural legal systems with contradictory laws adversely affect health by undermining national laws and international human rights commitments, and allowing harmful discriminatory practices to persist [12, 13]. Adherence to CRL in Nigeria has directly conflicted with international human rights commitments. Although Nigeria ratified CEDAW without reservation in 1985, the Nigerian National Assembly rejected the domestication of the CEDAW and it therefore is not part of Nigeria’s national legal framework, meaning its provisions are not justiciable or enforceable in Nigerian courts [49]. The CEDAW Committee has expressed “concern at contradictions and inconsistencies created by the application of statutory, customary and Sharia laws…particularly in the areas of marriage and family law…. It notes with concern the existence of discriminatory provisions within these sources of law with regard to marriage…lead[ing] to continuing discrimination against women” [49]. Additionally, in its Concluding Observations, the Convention on the Rights of the Child (CRC) Committee directed Nigeria to review the compatibility of customary laws with that of the values of the CRC, especially in regard to child marriage [50].

CRL’s effectiveness in undermining national health promotion policies is evident in other countries. In Zimbabwe, national civil law negates spousal approval requirements for medical treatment. However, customary law in parts of Zimbabwe requires a woman to obtain her husband’s consent for use of contraceptives and all other forms of medical treatment [51]. Another study noted that, despite signing onto the CRC, which sets the minimum age for marriage at 18, most countries in Sub-Saharan Africa have provisions allowing children to marry early under customary law or other circumstances [13]. Contradictory law regarding age of marriage in a country has been shown to be ineffective in reducing adolescent fertility rates [13]. Inconsistent legal frameworks, as in the case of child marriage laws, can lead to the exploitation of girls and increase the prevalence of teenage childbearing [13].

While our findings that CRL states have a higher median age at first birth and higher fertility rate than non-CRL states are closely linked to CRL through the association between child marriage, early childbearing, and number of births, other outcomes suggest an additional indirect relationship through norm-setting [52, 53], which was not examined here. CRL perpetuates harmful norms and practices that have an adverse effect at the population level, not just the individual level. For example, even though there is no CRL that formally limits access to contraception, antenatal care, and birthing facilities, our findings indicate that CRL negatively impacts their usage. In Nigeria, religion has largely been politicized, with male interpretations of religious morality used to control women’s bodies and sexuality [1, 48]. Scholars have noted that religious traditions in Northern Nigeria have often been used to legitimize the inferior status of women by emphasizing female submission, and expecting women to give birth to as many children as possible and obey their husband’s desires [1]. It has thus been hypothesized that CRL may influence SRH outcomes by increasing a woman’s socioeconomic dependence on her male partner, requiring that women be accompanied by their husbands away from home, limiting women’s decision-making or by upholding patriarchal norms [8, 42, 54]. Additional research is needed to better understand the indirect pathways through which norms under CRL impact SRH.

### Limitations

There are some limitations to the operationalization of contraceptive use, which included only married women. Future studies should seek to incorporate both married and unmarried sexually active women in estimates of contraceptive prevalence. Recognizing that laws and norms have a population-level effect, this analysis examined aggregate state-level data rather than individual-level data. This analytical approach resulted in a small number of observations. The confidence intervals for our models, however, were narrow, with p–values <0.001 for five of six unadjusted models and all five adjusted models; low statistical power was not a limitation. Use of state–level data did prevent us from accounting for more confounding variables beyond GDP; other confounders may explain some of the observed differences by CRL status. Future research should incorporate prospective individual-level data to further elucidate these findings, including the pathways by which CRL negatively impacts SRH.

## Conclusion

This analysis establishes a clear relationship between adherence to CRL and poor reproductive health outcomes for women and girls in Nigeria. CRL is often binding despite the existence of civil law or international standards aimed at improving health and gender equality. Countries should incentivize compliance with international standards on access to SRH and work towards the harmonization of laws that protect and promote access to SRH.
